# Influence of increased target dose inhomogeneity on margins for breathing motion compensation in conformal stereotactic body radiotherapy

**DOI:** 10.1186/1756-6649-8-5

**Published:** 2008-12-03

**Authors:** Anne Richter, Kurt Baier, Juergen Meyer, Juergen Wilbert, Thomas Krieger, Michael Flentje, Matthias Guckenberger

**Affiliations:** 1 Julius-Maximilians-University, Department of Radiation Oncology, Wuerzburg, Germany; 2 University of Canterbury, Department of Physics and Astronomy, Christchurch, New Zealand

## Abstract

**Background:**

Breathing motion should be considered for stereotactic body radiotherapy (SBRT) of lung tumors. Four-dimensional computer tomography (4D-CT) offers detailed information of tumor motion. The aim of this work is to evaluate the influence of inhomogeneous dose distributions in the presence of breathing induced target motion and to calculate margins for motion compensation.

**Methods:**

Based on 4D-CT examinations, the probability density function of pulmonary tumors was generated for ten patients. The time-accumulated dose to the tumor was calculated using one-dimensional (1D) convolution simulations of a 'static' dose distribution and target probability density function (PDF). In analogy to stereotactic body radiotherapy (SBRT), different degrees of dose inhomogeneity were allowed in the target volume: minimum doses of 100% were prescribed to the edge of the target and maximum doses varied between 102% (P102) and 150% (P150). The dose loss due to breathing motion was quantified and margins were added until this loss was completely compensated.

**Results:**

With the time-weighted mean tumor position as the isocentre, a close correlation with a quadratic relationship between the standard deviation of the PDF and the margin size was observed. Increased dose inhomogeneity in the target volume required smaller margins for motion compensation: margins of 2.5 mm, 2.4 mm and 1.3 mm were sufficient for compensation of 11.5 mm motion range and standard deviation of 3.9 mm in P105, P125 and P150, respectively. This effect of smaller margins for increased dose inhomogeneity was observed for all patients. Optimal sparing of the organ-at-risk surrounding the target was achieved for dose prescriptions P105 to P118. The internal target volume concept over-compensated breathing motion with higher than planned doses to the target and increased doses to the surrounding normal tissue.

**Conclusion:**

Treatment planning with inhomogeneous dose distributions in the target volume required smaller margins for compensation of breathing induced target motion with the consequence of lower doses to the surrounding organs-at-risk.

## Background

The process of treatment planning and dose calculation in radiation therapy is currently based on a three-dimensional (3D) patient model. Temporal changes of the patients' anatomy due to breathing motion for e.g. lung tumors are not easily accounted for [1]. The use of standard, population based margins has been shown to be insufficient for a considerable proportion of the patients [2]. Different strategies have been developed for quantification of breathing motion [3-5] and for integration of the motion information into the planning and treatment workflow [6-12].

Non-gated treatment of the patient breathing freely without tumor tracking is the most straightforward technique: the requirements on the patients' pulmonary function and cooperation are low, the irradiation is performed continuously resulting in short total treatment times and no specific technical equipment is required. However, precise assessment of breathing induced target motion and calculation of adequate safety margins are essential for this technique.

Four-dimensional respiratory correlated computed tomography (4D-CT) is considered as the standard to gain detailed information about range and pattern of breathing induced organ motion [13,14]. The next step is the integration of this temporal dimension into the process of dose calculation and treatment planning. A straightforward approach to approximate the effect of tumor motion on the dose distribution is convolving a static dose distribution with a matrix containing the probability density function (PDF) of the moving organ [15-22].

Engelsman et. al [23] used this method to investigate the influence of breathing induced motion of pulmonary tumors and to calculate margins for compensation of this uncertainty. The margin was described by the displacement of the 95% isodose in the blurred dose profile compared to the static dose profile. That study was analogous to current clinical practice in fractionated radiotherapy, where the target volume is treated with a homogenous dose distribution.

However, for some clinical indications like cranial and extracranial stereotactic radiotherapy, inhomogeneous dose distributions are commonly used. In pulmonary stereotactic body radiotherapy (SBRT) the degree of dose inhomogeneity in the target volume varies widely: doses are prescribed to the 50%, 60%, 65%, 80%, 85% or 90% isodose [24,25]. Motion of the target in such inhomogeneous dose distributions is expected to yield different results compared to simulations based on a homogenous dose distribution.

It was the aim of the current work to simulate the effect of tumor motion in inhomogeneous dose distributions. Inhomogeneous dose distributions were generated by field size reduction without intensity modulation. The convolution model was used to simulate the motion of a target in a homogeneous medium and the motion simulation was focused on respiratory motion while setup errors were ignored. The decrease of treatment dose in the target due to motion was evaluated and margins for compensation of breathing motion were calculated. Different degrees of dose inhomogeneity in the target for a single beam profile were used. The results of this study are expected to be of clinical relevance especially for conformal stereotactic body radiotherapy of pulmonary and intra-hepatic lesions.

## Methods

The investigations of this study are based on real patient data: ten consecutive patients (age 67 ± 6 years, female 3, male 7) treated with SBRT for early stage non-small cell lung cancer or pulmonary metastases. Written informed consent was obtained from all patients.

**Figure 1:**
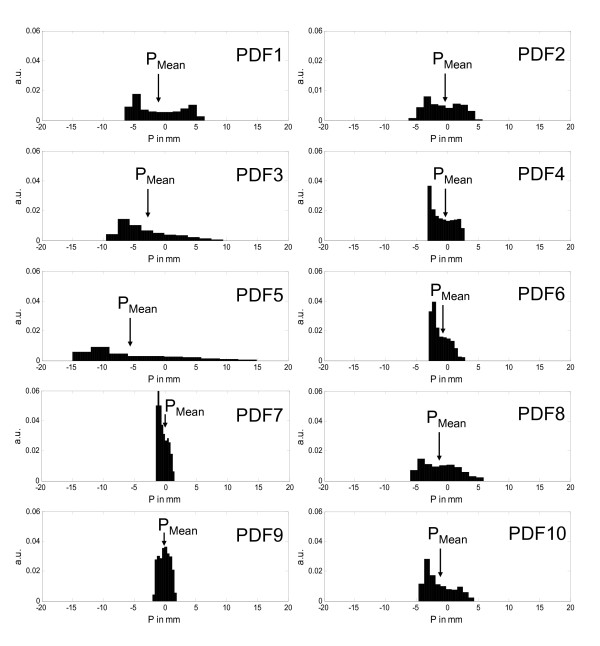
**Probability density functions of the pulmonary tumor for the 10 patients (exhalation and inhalation position have negative and positive numbers).** The zero position is the geometrical centre of the PDF and the mean target position (P_Mean_) is marked with an arrow.

For all ten patients a respiratory correlated 4D-CT study was acquired for treatment planning (Siemens Sensation Open; Siemens Medical Solutions, Erlangen, Germany). Details about 4D-CT image acquisition and image reconstruction have been described previously [24]. The motion range of the pulmonary target was evaluated after reconstruction of CT series in peak-inhalation and peak-exhalation. The motion of the tumor in the predominant direction was chosen for one-dimensional (1D) simulations: this was the superior-inferior direction in all patients. A pressure sensor placed in an elastic belt around the patient's abdomen generated the external breathing signal (Anzai AZ-733V; Anzai Medical Solutions, Japan). The external breathing signal recorded during acquisition of the 4D-CT study was the basis for the calculation of the tumor PDF; the range of the PDF was equated with the maximum motion range of the tumor measured in the 4D-CT (Figure 1).

The patient specific parameters are listed in table 1; breathing motion was quantified by the range of the PDF (A_Max_), standard deviation (Eq. 1) of the PDF (σ_PDF_) and the time-weighted mean target position (P_Mean_). The standard deviation of the motion signal P was calculated using the position sample (P_i_), the mean target position (P_Mean_) and the number of elements in the sample (n).

**Table 1:** Overview of patient data derived from 4D-CT imaging and the external breathing signal

**Patient**	**Tumor Ø** **(mm)**	**A_Max_** **(mm)**	**σ_PDF_** **(mm)**	**P_Mean PDF_** **(mm)**
**1**	18	11.5	3.8	-1.0
**2**	18	11.1	2.8	-0.6
**3**	12	17.4	4.2	-3.1
**4**	25	4.9	1.8	-0.7
**5**	40	26.8	7.1	-5.6
**6**	32	5.5	1.4	-1.2
**7**	27	3.0	0.8	-0.4
**8**	15	10.9	2.9	-1.2
**9**	10	3.6	0.9	-0.1
**10**	8	7.8	2.3	-1.3

Abbreviations: range of the probability density function (PDF) A_Max_, standard deviation of the PDF σ_PDF_, time-weighted mean target position P_Mean_: this position is defined as the distance from the geometrical centre of the PDF

(1) $$\sigma_{\text{PDF}}=\sqrt{\frac{1}{n-1}\sum_{i=1}^{n}{\left(P_{i}-P_{Mean}\right)}^{2}}$$

A 1D convolution model was used to investigate the dosimetric influence of breathing induced tumor motion. The model was implemented in Matlab™ (The MathWorks, Inc., Natick, MA, USA) and allowed the variation of the following parameters: dose profile, field size, target size, motion range and shape of the PDF.

Dose profiles were generated by convolving an intensity profile with a pencil beam kernel while a homogeneous medium was assumed [26]. The intensity profile was a square profile simulating an open field shape. To ensure the correctness of the computed dose profiles, a comparison with measured profiles in a water phantom was performed with the following parameters: field size 24 × 24 mm, depth 50 mm, machine Elekta Synergy S equipped with the BeamModulator with 4 mm leaf width (Elekta Oncology Systems, Crawley, UK) and photon energy 6 MV. The calculated and measured dose profiles were in good agreement with 0.07% mean error normalized to maximum value. The resolution of the simulated dose profiles was 0.1 mm.

**Figure 2:**
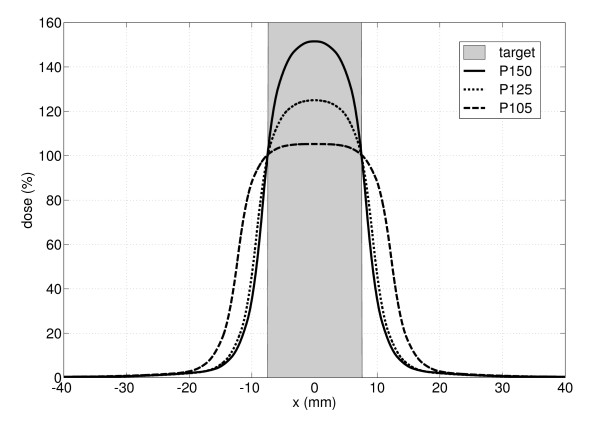
**Illustration of three different dose prescriptions P105, P125 and P150.** The minimum dose was set to 100% at the edges of the target with maximum doses of 105%, 125% and 150%.

Assuming no breathing motion, a minimum dose of 100% was prescribed to the edge of the static target in the time-weighted average position P_Mean_. The centre of the target in this time-weighted average position was the isocentre as suggested by Wolthaus et al. [27]. According to the SBRT, different degrees of dose inhomogeneity in the target volume were simulated. The maximum dose in the tumor was varied between 102% and 150%. The dose inhomogeneity was achieved by variation of the field size only, no step-and-shoot segments for intensity modulation were used. Figure 2 displays the prescription types P105, P125 and P150 while P105 corresponds to a plan with a minimum dose of 100% and a maximum dose of 105% within the target volume.

The time accumulated dose profile *D*_*acc *_was calculated by convolving the static reference dose profile *D*_0_(*x*) with the variation kernel *PDF*(*x*).

(2) $$D_{acc}=\int D_{0}(x-x^{\prime })•PDF(x)dx^{\prime }$$

**Figure 3:**
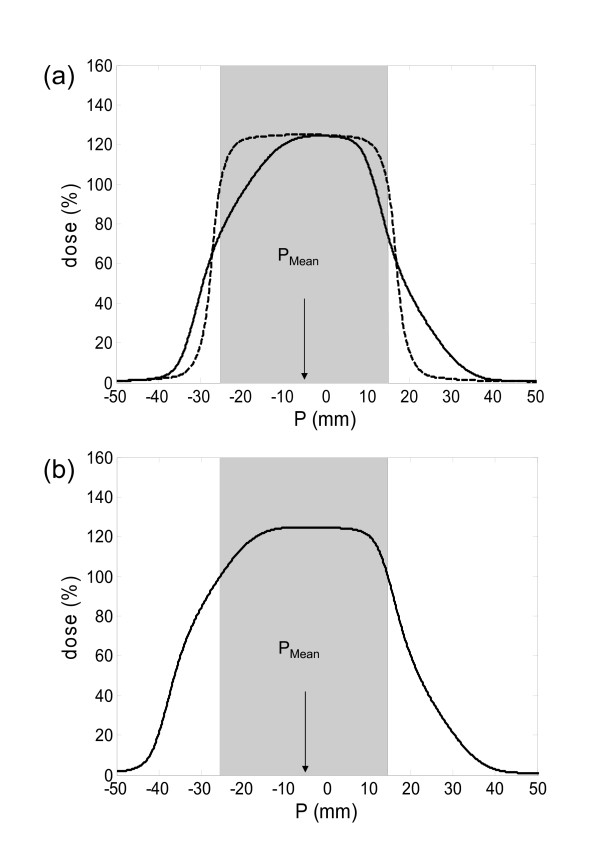
**Workflow of convolution simulation for patient #5 for prescription type P125 (the shaded area represents the target in the time weighted mean position 5.6 mm).** (a) The dose to the target without (dashed line) and with consideration of motion (solid line). (b) The accumulated dose with an additional margin of 4.9 mm for motion compensation.

This resulted in a blurring of the dose gradient and consequently reduced the accumulated dose to the target volume (Figure 3a). For quantification of the motion effect, the dose to the static target was compared with the dose after motion simulation. The minimum dose to the target (D_Min_) was considered as the treatment dose. Then, field sizes were symmetrically enlarged in steps of 0.1 mm until the dose reduction due to breathing motion was completely compensated (Figure 3b). The position of the dose profile was shifted and optimized until the tumor received equal doses to the edges. A margin was defined as the increase in field size in one direction; the total increase in field size is consequently twice the one-sided margin. This target volume concept for compensation of breathing motion was termed as "optimization of the surrounding isodose" (OSI concept). Additional blurring of the dose profile due to setup errors were not considered in this work.

Additionally, the influence of motion on the dose to the region surrounding the tumor (organs-at-risk lung or liver) was investigated. This region corresponds to the pulmonary/hepatic tissue superior and inferior to the target in a 1D patient model, because target motion in superior-inferior direction was modelled. In real patient treatment, a significant dose is delivered to healthy tissue in the entrance and exit regions of the beam (axial direction) assuming a predominant coplanar beam set-up. This is not considered in a 1D model. Consequently, doses to the surrounding organ-at-risk were transformed into a 2D model in a sagittal plane: with a coplanar opposing field design, the dose distribution in superior-inferior direction was extrapolated in anterior-posterior direction. Doses in a 2D sagittal plane with extensions of 15 cm in superior-inferior and 12 cm anterior-posterior direction were calculated, respectively; the dose inside the target (equal tumor size in superior-inferior and anterior-posterior direction) was excluded for dose calculation to this organ-at-risk. As the mean lung dose was shown to be predictive for radiation induced pulmonary toxicity [28], the mean dose in this region was calculated (D_Mean Surr_).

The simulation in this study was divided into four parts to analyse the influence of the parameters motion range, dose inhomogeneity and motion pattern, separately.

### Range of breathing motion

The first part of the study systematically quantified the influence of the motion range on the dose to target. For a single breathing pattern (PDF#1) and a target size of 20 mm, the motion range A_Max _was varied ranging from 2 mm to 30 mm. These simulations were performed based on dose prescriptions P105, P125 and P150, which are most frequently used in pulmonary and hepatic SBRT. Margins necessary for compensation of the dose loss due to breathing motion were calculated.

### Breathing pattern

The second part of the study systematically evaluated the influence of the breathing pattern. For one single target size of 20 mm and A_Max _of 15 mm the breathing pattern was varied with the ten clinically observed PDFs. The simulation was performed for P105, P125 and P150 and margins for motion compensation were calculated.

### Dose inhomogeneity

The third part of the study systematically evaluated the influence of the dose inhomogeneity in the target on margins necessary for compensation of breathing motion. Dose prescriptions from P102 to P150 were simulated. This was done exemplary for three different patients (#1, #5, #8) and the corresponding tumor PDF, A_Max _and tumor size. Simulations for P105, P125 and P150 were performed using all ten patient data (Table 1). Additionally, the influence of dose inhomogeneity on D_Mean Surr_, the mean dose to the tumor surrounding region, was evaluated.

### ITV versus OSI target volume concept

The internal target volume (ITV) concept was evaluated as this is most commonly used in SBRT treatment: the ITV encompassed the target during the whole breathing cycle and the 100% dose was prescribed to cover the ITV. Results of the OSI and ITV concepts were compared with dose prescriptions of P105, P125 and P150.

## Results

### Range of breathing motion

The first simulation evaluated the influence of the motion range A_Max _on the dose to the target. An increasing range of breathing motion resulted in lower D_Min _to the target. For motion compensation, the optimal isocentre position for equal doses at both edges of the target volume agreed closely with the time-weighted mean target position for 8 of 10 patients. Differences of 0.9 and 2.1 mm were observed for patients 3 and 5. These patients showed large motion amplitudes of 17 and 27 mm with highly asymmetrical breathing patterns.

**Figure 4:**
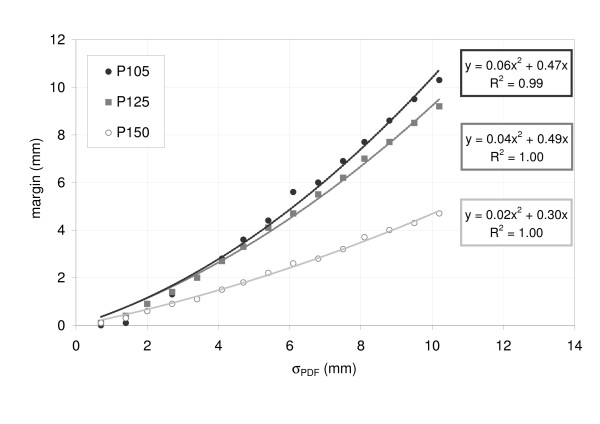
Margins for compensation of breathing induced dose loss to the target as a function of target motion (σ_PDF_) for PDF#1.

A polynomial relationship of second order was found between σ_PDF _and the margin size: for target motion between 2 mm and 30 mm the margin increased quadratically (coefficients of determination (COD) 0.99). For P105, a margin of 2 mm was needed for A_Max _= 10 mm and σ_PDF _= 3.4 mm. If the motion (A_Max _and σ_PDF_) was twice as large, the margin size increased to 6 mm. Results for the three different prescriptions are shown in Figure 4. A higher degree of dose inhomogeneity resulted in smaller margins for motion compensation. For example, to compensate the dose loss due to target motion of σ_PDF _= 6.8 mm and A_Max _= 20 mm, margins of 6 mm, 5.5 mm and 2.8 mm were necessary for P105, P125 and P150, respectively. Isodose levels close to the 50% ideally don't move at all under dose blurring. In Figure 3 it was demonstrated, that especially smaller isodose (e.g. 65%) levels of the accumulated dose profile were shifted less than higher isodose levels (100%).

### Breathing pattern

**Figure 5:**
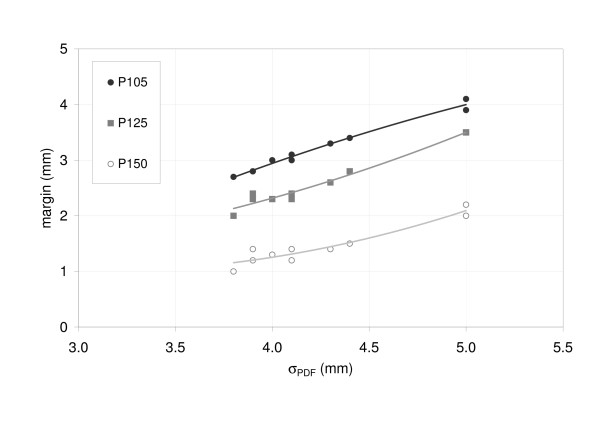
Margins size as a function of the standard deviation of target motion (σ_PDF_) for the ten clinically observed breathing patterns; motion range (15 mm) and target size (20 mm) remained constant in this experiment.

The influence of different breathing patterns on the margin size was investigated while A_Max _was maintained constant at 15 mm: due to differences in the shapes of the PDFs, the σ_PDF _ranged between 3.8 mm and 5 mm. For the three prescription types (P105, P125 and P150), the data showed larger margins for increased σ_PDF_. Despite a constant A_Max_, the margins for motion compensation varied between 2.7 mm and 4.1 mm for P105. Again, larger margins were needed for P105 than for P150 (Figure 5). These results show that σ_PDF _is a more robust parameter to describe the target motion and PDF shape than A_max _which characterizes only the width of target motion.

### Dose inhomogeneity

**Figure 6:**
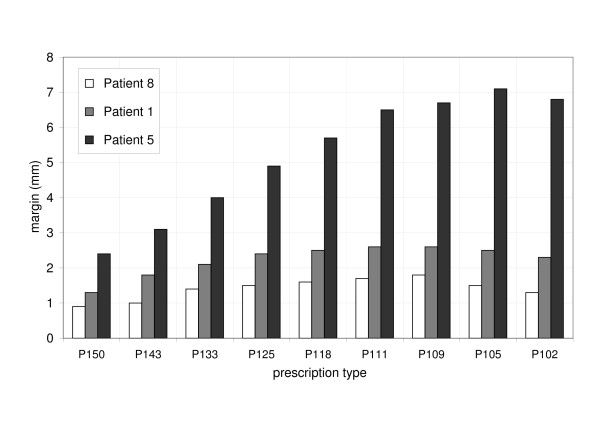
Margins for compensation of breathing induced dose loss to the target as a function of the dose prescription for three particular patients (#8, #1, #5).

The third simulation evaluated the influence of dose inhomogeneity in the target on margins for compensation of breathing motion. The dose prescription was varied from P102 to P150, margins were increased until the minimum dose to the target was 100%. The results are illustrated in Figure 6. Margins were smallest for P150 and largest for P109 and P105: for A_Max _of 11.5 mm and target size of 18 mm (patient #1), margins of 1.3 mm, 2.4 mm and 2.5 mm were required for P150, P125 and P105, respectively. The advantage of smaller margins for increased target dose inhomogeneity was observed for all three patients but the benefit was more obvious for large tumor motion.

The investigation of different degrees of dose inhomogeneity in the target showed generally an increase in margin size for more homogeneous target dose. Depending on the shape of the PDF, we observed a small decrease in margin size from P105 or P109 to P102 (Figure 6). The decrease was caused by convolving a gaussian shaped profile with a non symmetric PDF. Before convolution, the curvature of the static profile was monotonically decreasing from 66% (P150) to 98% (P102) isodose level. This was not the case for the accumulated dose profile which was deformed depending on the shape of the PDF. The distance of the prescription isodose level between static and accumulated profile was largest for P105 and P109 which resulted in the maximum compensation margins. The distance decreased for larger prescription isodose levels and therefore the necessary margin size was decreasing as well.

**Figure 7:**
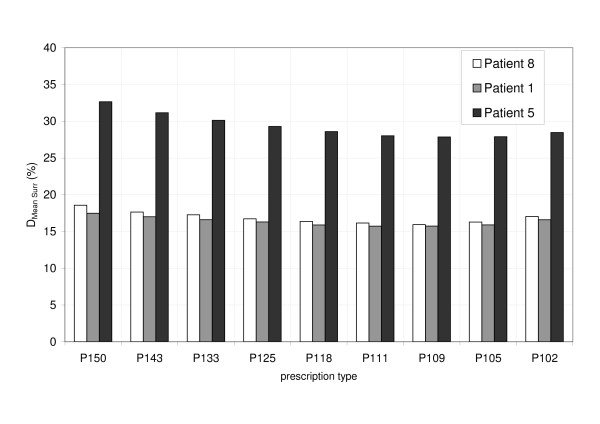
Accumulated mean dose to the surrounding area D_Mean Surr _as a function of the dose prescription for three patients (#8, #1, #5).

Figure 7 shows the mean dose to the organ-at-risk surrounding the target as a function of dose inhomogeneity from P150 to P102 for three patients. Motion compensation with dose inhomogeneities from P105 to P118 resulted in the lowest mean dose to surrounding organ-at-risk. Despite smaller safety margins in superior-inferior direction for larger dose inhomogeneity than P118, the mean dose to the surrounding organ-at-risk increased due to higher doses in the entrance and exit region of the radiation beams.

**Figure 8:**
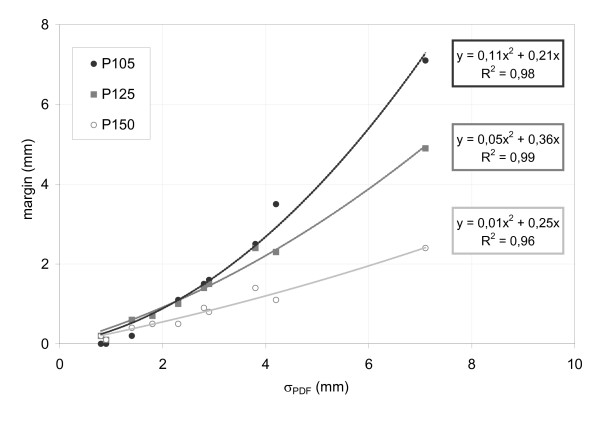
Margins for compensation of breathing induced dose loss to the target for all ten clinically observed patients.

The effect of smaller safety margins with increased dose inhomogeneity was validated for all ten patients (Figure 8).

### ITV versus OSI target volume concept

The comparison of motion compensation using the ITV concept and the OSI concept showed differences for margins, target dose and D_Mean Surr _(Table 2). Margins for compensation of breathing motion were smaller based on the OSI concept compared to the ITV concept. As a consequence, the ITV concept over-compensated the effects of breathing motion with higher than planned doses to the target: the ITV concept increased D_Min _to the target by 3.6 ± 0.7%, 17.8 ± 4.0%, 37.2 ± 8.9% for P105, P125 and P150, respectively. Simultaneously, D_Mean Surr _was increased for the ITV concept compared to the OSI concept.

**Table 2:** Mean differences between internal target volume concept (ITV) and optimization of the surrounding isodose (OSI)

	**P105**	**P125**	**P150**
	
**Δ D_Min _in %**	3.6 ± 0.7	17.8 ± 4.0	37.1 ± 8.9
**Δ Margin in mm**	3.3 ± 1.6	3.6 ± 2.3	4.3 ± 3.0
**Δ D_Mean Surr _in %**	7.8 ± 3.0	6.4 ± 4.3	4.9 ± 3.1

Abbreviations: absolute increase in minimum target dose Δ D_Min_, absolute increase in margin size Δ Margin, absolute increase in mean dose to surrounding region Δ D_Mean Surr_

## Discussion

This study used a one-dimensional convolution model to simulate the effects of breathing induced target motion in radiotherapy treatment. We allowed dose inhomogeneity in the target, which is considered as the standard in SBRT treatment of intra-hepatic and intra-pulmonary tumors [29]. The dose inhomogeneity was achieved by changing field size and renormalization of the one-beam profile. No step-and-shoot segments were used to achieve dose inhomogeneity. Consequently, the results are of interest especially for conformal SBRT. However, the concept of inhomogeneous dose distributions for mobile target volumes may gain relevance for radiotherapy treatment in general.

The time-weighted mean tumor position was selected as the isocentre in this study. This set-up ensured, that minimum doses after consideration of breathing motion without addition of extra margins were identical at both "edges" of the target for nearly all patients. As a consequence, the addition of margins resulted in equal dose compensation to 100% at both edges of the target. A comparable concept with treatment planning based on the mean tumor position has been described by Wolthaus et al. [27]. Consequently, the time-weighted mean target position is considered as the appropriate reference for treatment planning of target volumes with clinically large breathing motion.

### Influence of PDF shape and target dose inhomogeneity on margin size

The shape of the probability density function – i.e. how long does the target stay at which position – influenced margins for motion compensation. The correlation between safety margins and maximum range of tumor motion was suboptimal: different margins were calculated for different shapes of the tumor PDF despite identical motion range. The maximum motion range describes the width of motion but not the shape of motion pattern. In all settings the standard deviation of the PDF was closely correlated to safety margins. This is in agreement to the findings of Jiang who showed the dose loss due to motion depends on the steepness of the dose profile and the shape of the PDF which is described by the standard deviation [30].

We observed a quadratic relationship between margins for compensation of motion induced dose loss and the motion of the tumor expressed by the standard deviation. The quadratic relationship between safety margins and tumor motion was observed for all investigated degrees of dose inhomogeneity in the target volume but was most pronounced for P105 and least for P150, where a rather linear relationship between motion and margins was observed. A similar quadratic relationship has been described by Engelsman et al. and Sonke et al. [23,31]; these studies were limited to homogeneous dose distributions, which is the standard in conventionally fractionated radiotherapy.

The margins for compensation of breathing motion were smaller, if a higher degree of dose inhomogeneity was allowed in the target volume. Thus increased dose inhomogeneity reduced the absolute irradiated volume of the surrounding organ-at-risk. However, increased dose inhomogeneity in the target volume was associated with increased doses to the target surrounding organ-at-risk in the entrance and exit beam regions. These contrary effects of dose inhomogeneity showed an optimum with minimum mean dose to the surrounding organ-at-risk for dose prescriptions between P105 and P118. The risk of normal tissue injury should be minimal in this region of dose inhomogeneity with simultaneously identical minimum doses to the target.

Such a benefit of inhomogeneous dose distributions for treatment of pulmonary targets has been described by Engelsman et al. [32]: an iso-NTCP increase of the tumor control probability was observed for reduced field sizes with higher dose inhomogeneity in the target. In accordance to our study, an optimal dose inhomogeneity in the target volume was observed. Data from that study were based on phantom measurements with simulated, regular breathing motion. Results of our study were in agreement, and confirmed this concept for inhomogeneous dose distributions based on irregular breathing motion of real patient data.

The internal target volume concept is most frequently used in clinical practice: the amplitude of breathing induced target motion, target positions in end-inhalation and end-exhalation, define the size of target volume. The comparison of the ITV and OSI concept ascertained that the ITV concept over-estimated the influence of breathing motion [33] with unnecessary large margins. Increased doses to the normal tissue and higher as planned doses to the target are consequences.

### Limitations of the study

Though convolution calculations are an accepted model for investigation of spatial and temporal uncertainties in radiotherapy, limitations of this method are well known [17,18] and some issues need to be considered for correct interpretation of results from this study. We did not aim to give numerical data for motion compensation which can be directly transferred into clinical practice. The focus of our work was to show the relation between motion compensation and a potential benefit of dose inhomogeneity.

Tissue inhomogeneities, differences in density between the solid tumor and the surrounding normal tissue are not considered. This may be of minor relevance for SBRT treatment of intra-hepatic targets. However, for intra-pulmonary tumors a decreased dose deposit in the pulmonary tissue of lower density and in the periphery of the solid tumor is well known [34]. We have recently shown, that this effect significantly influences the 4D dose calculation for pulmonary targets [33]: motion of the tumor and of the surrounding normal tissue resulted in a variant, non-static dose distribution during the breathing cycle. Modelling of such effects with a convolution model is not possible. Despite this limitation, we decided to perform this study with the convolution method, because this approach allowed systematic analysis of the parameters dose profile, field size, target size, motion range and shape of the PDF. Such a large number of simulations would not have been possible with realistic four-dimensional treatment planning.

The simulations in this study were limited to one spatial dimension. In all ten patients, breathing motion of the target was largest in superior-inferior direction and consequently, motion in this direction was simulated. However, breathing motion in other than superior-inferior direction is well known [1]. With predominantly co-planar beam set-up the dose gradient is usually less steep in anterior-posterior and left-right direction compared to superior-inferior. Subsequently, margins for compensation of motion in these directions are expected to be even smaller compared to the margins calculated in this study [19,30,35].

The external breathing signal was used as surrogate for generation of the tumor PDF; the range of tumor motion was evaluated in respiratory correlated CTs. This method of PDF generation was chosen to obtain realistic tumor PDFs based on several breathing cycles. It is well known that the correlation between external surrogate and internal target motion is inconsistent [8,10]. However, internal and external motion is usually best correlated for target motion in superior-inferior direction, which was simulated in our study. Additionally, phase shifts between internal and external motion are truly relevant for 4D-CT reconstruction and gated radiotherapy, but such phase shifts would have no influence on the results of our study. Consequently, this procedure is not considered as relevant for interpretation of results of our study: the benefit of increased dose inhomogeneity in terms of reduced safety margins for motion compensation was observed for all patients with various shapes and ranges of the tumor trajectories.

The simulation was based on one respiratory correlated CT acquired for treatment planning. Inter-fractional changes of the patients' breathing motion and breathing pattern are not considered. However, several studies have shown that one single respiratory correlated planning CT study was representative for the duration for a SBRT and conventionally fractionated treatment course [36-38].

The PDF binning was set to 10 bins for each PDF which resulted in different PDF resolutions in space depending on the maximum motion amplitude. The choice was based on the fact, that in clinical routine always the same number (8–10) of respiration phases is chosen for reconstruction of the 4D-CT.

### Clinical consequences

Optimal sparing of the organ-at-risk surrounding the target was achieved for dose prescriptions P105 to P118. For higher dose prescriptions, the effect of smaller safety margins was overcompensated by increased doses in the entrance and exit regions of the radiation beams. The mean dose to the surrounding organ-at-risk was the basis for this conclusion. Recently however, several studies have shown that very small doses between 5 Gy and 13 Gy [39-41] are most predictive for radiation induced pneumonitis. If this were true the pulmonary tissue in the entrance and exit regions of the radiation beams would be "sacrificed" regardless of the dose homogeneity in the target volume. In such a scenario, sparing of lung tissue in superior-inferior direction would be more clinically relevant with the consequence of a benefit for dose inhomogeneities greater than P125.

Two recent studies in the literature described four-dimensional treatment planning using respiratory correlated CT. These studies used the ITV target volume concept for SBRT of pulmonary [33] and liver tumors [42] and a comparison between dose inhomogeneity of P125 and P150 was performed in both studies. These studies confirmed that an increased dose inhomogeneity resulted in increased doses to the target without a significantly increased dose the surrounding organs-at-risk. These conclusions support the findings in our study: the concept of inhomogeneous dose distributions should be developed further in the future.

Margins in our study were calculated for compensation of intra-fractional uncertainties of the target position, only. Other uncertainties in the process of treatment planning and treatment delivery need to be compensated with extra margins. Uncertainties in image acquisition and contouring of mobile targets require additional margins. Even high-precision stereotactic patient positioning was shown to result in large errors of the target position especially because of base-line shifts of the tumor independently from the bony anatomy [37,38,43-45]: decreased tumor control probability and increased risk of damage to adjacent critical structures are consequences [46,47]. The effects of such set-up errors on the dose to the target are expected to be more pronounced for treatment planning with a significant dose inhomogeneity in the target volume and consequently steep dose gradients close to the tumor. Image-guidance has been effective for compensation of these inter-fractional uncertainties and is considered as prerequisite for such high-precision radiotherapy [11,36,48].

It is essential, that such aggressive protocols with increased maximum doses need to be balanced with clinical, patient specific factors. Whereas high dose inhomogeneity might be clinically acceptable for targets in the periphery of the lung, one would be cautious for centrally located targets [49]. However, it is clear that the way of dose prescription gives an additional degree of freedom in treatment planning to the physician and the physicist.

## Conclusion

It was demonstrated that the time-weighted mean tumor position and the standard deviation of breathing induced target motion are the most relevant parameters in radiotherapy treatment planning of mobile targets. A quadratic relationship between motion and margins for compensation of dose loss was observed for various degrees of dose inhomogeneity within the target volume. A margin reduction was possible by allowing an inhomogeneous dose distribution in the target. The internal target volume concept over-compensated breathing motion with the consequence of higher than planned doses to the target and increased dose to the lung.

## Competing interests

The authors declare that they have no competing interests.

## Authors' contributions

All authors read and approved the final manuscript. AR designed the study and the analysis, performed the simulations and revised the manuscript. KB, JW, TK and MF participated in the study design and revised the manuscript. JM participated in the study design and analysis and revised the manuscript. MG designed the study and participated in the study design and revised the manuscript.

## Pre-publication history

The pre-publication history for this paper can be accessed here:


